# Personalized‐induced neural stem cell therapy: Generation, transplant, and safety in a large animal model

**DOI:** 10.1002/btm2.10171

**Published:** 2020-07-15

**Authors:** Hunter N. Bomba, Kevin T. Sheets, Alain Valdivia, Simon Khagi, Laura Ruterbories, Christopher L. Mariani, Luke B. Borst, Debra A. Tokarz, Shawn D. Hingtgen

**Affiliations:** ^1^ Division of Pharmacoengineering and Molecular Pharmaceutics, UNC Eshelman School of Pharmacy The University of North Carolina at Chapel Hill Chapel Hill North Carolina USA; ^2^ Department of Neurosurgery The University of North Carolina at Chapel Hill Chapel Hill North Carolina USA; ^3^ Lineberger Comprehensive Cancer Center The University of North Carolina at Chapel Hill Chapel Hill North Carolina USA; ^4^ Department of Clinical Sciences, College of Veterinary Medicine North Carolina State University Raleigh North Carolina USA; ^5^ Comparative Medicine Institute North Carolina State University Raleigh North Carolina USA; ^6^ Department of Population Health and Pathobiology, College of Veterinary Medicine North Carolina State University Raleigh North Carolina USA

**Keywords:** glioblastoma, neural stem cells, stem cell delivery

## Abstract

In this study, we take an important step toward clinical translation by generating the first canine‐induced neural stem cells (iNSCs). We explore key aspects of scale‐up, persistence, and safety of personalized iNSC therapy in autologous canine surgery models. iNSCs are a promising new approach to treat aggressive cancers of the brain, including the deadly glioblastoma. Created by direct transdifferentiation of fibroblasts, iNSCs are known to migrate through the brain, track down invasive cancer foci, and deliver anticancer payloads that significantly reduce tumor burden and extend survival of tumor‐bearing mice. Here, skin biopsies were collected from canines and converted into the first personalized canine iNSCs engineered to carry TNFα‐related apoptosis‐inducing ligand (TRAIL) and thymidine kinase (TK), as well as magnetic resonance imaging (MRI) contrast agents for in vivo tracking. Time‐lapse analysis showed canine iNSCs efficiently migrate to human tumor cells, and cell viability assays showed both TRAIL and TK monotherapy markedly reduced tumor growth. Using intraoperative navigation and two delivery methods to closely mimic human therapy, canines received autologous iNSCs either within postsurgical cavities in a biocompatible matrix or via a catheter placed in the lateral ventricle. Both strategies were well tolerated, and serial MRI showed hypointense regions at the implant sites that remained stable through 86 days postimplant. Serial fluid sample testing following iNSC delivery showed the bimodal personalized therapy was well tolerated, with no iNSC‐induced abnormal tissue pathology. Overall, this study lays an important foundation as this promising personalized cell therapy advances toward human patient testing.

## 1 INTRODUCTION

The notoriously hard‐to‐treat brain tumor glioblastoma (GBM) consists of highly infiltrative cells. Standard treatment for patients with GBM consists of maximal tumor surgical resection followed by chemoradiotherapy. However, obtaining clean tumor margins is difficult, and residual tumor cells invade normal brain tissue, creating distant tumor foci.^1^ Curtailing tumor recurrence with systemic therapies has seen limited success as the blood–brain barrier (BBB) tightly regulates the passage of molecules from systemic circulation to the brain parenchyma, which prevents many chemotherapies from reaching infiltrative GBM cells.^2^ Treatment advances for GBM must combat tumor recurrence by targeting the infiltrative cells that remain after standard therapy.

Induced neural stem cells (iNSCs) generated by the direct transdifferentiation of fibroblasts from a patient's own skin have recently shown promise as drug carriers for GBM due to their innate tumor tropism and low immunogenicity.^3^ These tumor‐seeking cells are genetically engineered to express cytotoxic proteins as they migrate toward invasive cancer cells. Early testing of iNSC therapy in preclinical models has demonstrated the effectiveness of iNSCs generated from human donors against human xenografts of GBM in athymic nude mice.^3^, ^4^, ^5^ Although the athymic mouse model recapitulates many aspects of human tumor resection surgery, it is understandably limited in its ability to test iNSC doses on par with what would be administered to a human patient as well as its ability to elucidate any potential adaptive immune reactions. Additionally, a small animal model cannot accurately mimic the size of the surgical cavity or fluid volumes that iNSCs may be delivered into during initial human patient testing. A large animal model is needed to bridge the gap between mouse models and a first‐in‐human Phase I clinical trial.

Canine models can be used to bridge this translational gap, as gliomas occur spontaneously in canines with phenotypes and genetic mutations similar to humans.^6^, ^7^ While this study focused on testing the safety and toxicity of iNSCs in healthy, nontumor‐bearing canines, this was an important consideration when selecting an animal model that would be compatible with future efficacy studies. Despite the potential of this large‐scale model, the generation of iNSCs from canine skin has not yet been reported. Additionally, the exploration of the delivery, dosing, and safety of autologous or allogeneic tumor‐homing cell therapies has primarily focused on the murine model and is extremely limited in large animal models.^8^, ^9^, ^10^ As such, we sought to demonstrate the feasibility of manufacturing, as well as the safety and toxicity of autologous therapeutic iNSCs using healthy canine patients. The iNSCs used in this study carry two therapeutic agents. The first therapeutic agent is TNFα‐related apoptosis‐inducing ligand (TRAIL), a constitutively expressed protein that is continuously secreted into the extracellular space. TRAIL diffuses to nearby cells but initiates caspase‐mediated apoptosis by engaging death receptors upregulated on cancer cells.^11^ Importantly, TRAIL has been well tolerated and has shown negligible off‐target toxicities in normal cells in both preclinical and clinical studies.^12^, ^13^, ^14^, ^15^ The second constitutively expressed protein is the enzyme thymidine kinase (TK). TK remains inactive until administration of its nontherapeutic prodrug substrate valganciclovir (VGCV). VGCV is first hydrolyzed into ganciclovir (GCV) in the liver and intestine, and subsequently, TK expressed by iNSCs phosphorylates circulating GCV into cytotoxic ganciclovir triphosphate (GCV‐TP). Finally, GCV‐TP inhibits DNA polymerase, consequently killing both the iNSC and nearby tumor cells via the bystander effect.^16^, ^17^ TK‐VGCV therapy exhibits limited toxicity on normal brain cells due to their quiescent state.^18^, ^19^, ^20^

Using our early‐stage studies in murine models as a guide, we demonstrate the production, safety, and toxicity of autologous therapeutic iNSCs transdifferentiated from canine skin for the first time. Healthy, purpose‐bred canines were successfully administered two dose levels of autologous iNSCs using two clinically relevant delivery methods: intracerebroventricular (ICV) infusion and scaffold encapsulation.^21^, ^22^ We further demonstrate the safety and minimal toxicity signals in the canine model using magnetic resonance imaging (MRI), blood, urine, cerebrospinal fluid (CSF), neurological assessment, and histopathology. These promising results pave the way for future efficacy studies in a spontaneous canine glioma model as well as human clinical trials.

## 2 RESULTS

### 2.1 Generation of canine iNSCs: Isolation, expansion, and conversion

**FIGURE 1:**
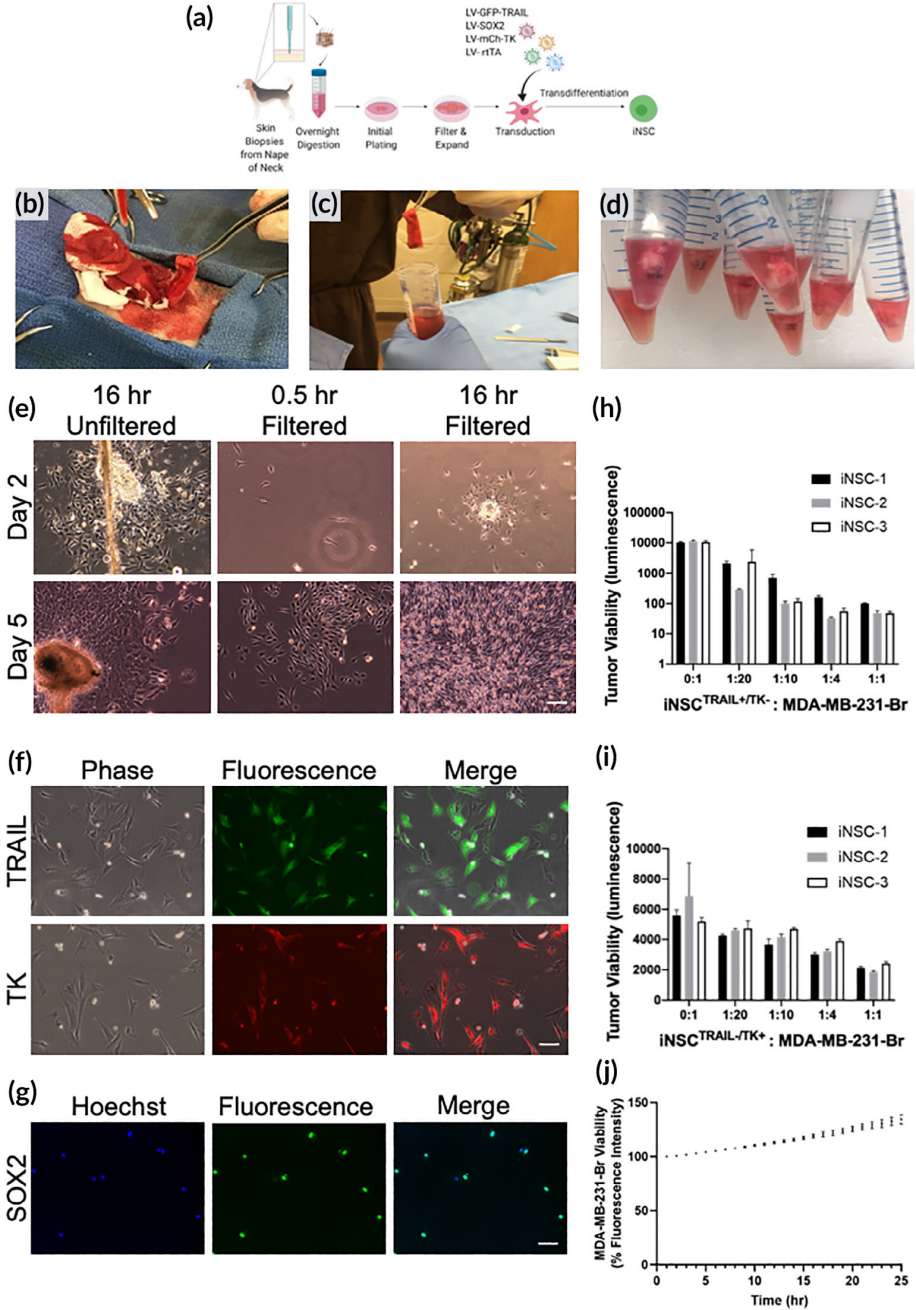
Murine proof‐of‐concept studies. (a) Schematic depicting fibroblast isolation, transduction, and transdifferentiation procedures. (b) Skin biopsy harvest from cadaver canine. (c,d) Skin samples in digestion media. (e) Cultures of primary canine fibroblasts 16 hr after plating and prior to filtering, 30 min after filtering, and 16 hr after filtering. Scale bar 100 μm. (f) Fluorescence imaging confirming TRAIL expression in fibroblasts. Scale bar 100 μm. (g) Immunofluorescence imaging of transdifferentiated fibroblasts to confirm SOX2 expression. Scale bar 50 μm. (h) Quantification of iNSCTRAIL+/TK− and MDA‐MB‐231‐Br 42 hr coculture assay. (i) Quantification of iNSCTRAIL−/TK+ and MDA‐MB‐231‐Br 72 hr coculture assay. (j) Quantification of iNSCTRAIL−/TK− and MDA‐MB‐231‐Br 25 hr coculture assay. All data presented as mean ± *SD*. iNSCs, induced neural stem cells; TK, thymidine kinase; TRAIL, TNFα‐related apoptosis‐inducing ligand

Previously, we have shown the ability to convert mouse and human fibroblasts into tumor‐homing iNSCs.^3^ Using these studies as a guide, we first explored the feasibility of generating personalized canine iNSCs (Figure 1a). First, full thickness (epidermis and dermis) skin biopsies were collected from donor canines. Once the animals were anesthetized, a skin biopsy was isolated from the base of the neck and placed into a tube containing collection media (Figure 1b,c). To isolate the fibroblasts, skin biopsies were manually diced into approximately 6 mm pieces (Figure 1d) and placed in digestion media containing a low concentration of collagenase, thus enabling long dissociation conditions without negative impacts on cell viability.^23^, ^24^ Following dissociation, the tissue pieces were seeded in culture plates containing growth media, and fibroblasts were allowed to grow for 72 hr. Once fibroblasts had expanded, the cells were trypsinized, filtered to removed unwanted solid tissue, and the remaining fibroblasts were allowed to proliferate rapidly. As shown in Figure 1e, we observed robust outgrowth of fibroblasts from all biopsies. While fibroblasts initially proliferated reproducibly after each passage, cell senescence was observed as early as passage 12 for some fibroblast lines (data not shown). This timeline dictated the fibroblast transduction and transdifferentiation processes. As we have shown previously, we used the SOX2 transcription factor to and transdifferentiation media to convert fibroblasts into tumor‐homing iNSCs.^3^ To explore the potential of converting canine fibroblasts into canine iNSCs, we transduced the canine cells with lentiviral vectors encoding reverse tetracycline‐controlled transactivator (rtTA) and SOX2, as well as the therapeutic and optical reporters mCherry‐TK, and enhanced green fluorescent protein (eGFP) fused to TRAIL. Following transduction and 5 days of transdifferentiation with culture in the presence of doxycycline, >85% of cells expressed TRAIL and >95% of cells expressed TK as demonstrated by eGFP and mCh fluorescence, respectively (Figure 1f). Additionally, immunofluorescence staining showed nearly all cells expressed transgenic SOX2, suggesting efficient transduction and conversion to the iNSC phenotype (Figure 1g). In total, three fibroblast cell lines (biological replicates) were established and labeled iNSC‐1, ‐2, and ‐3, respectively (Table 1).

**TABLE 1:** Abbreviations of cell types used

Cell type	Abbreviation
Neural stem cells	NSC
Induced NSCs generated from skin via direct transdifferentiation	iNSC
iNSCs expressing both TRAIL and TK but not exposed to prodrug ganciclovir	iNSC^TRAIL+/TK−^
iNSCs expressing both TRAIL and exposed to prodrug ganciclovir	iNSC^TRAIL+/TK+^

Abbreviations: iNSCs, induced neural stem cells; TK, thymidine kinase; TRAIL, TNFα‐related apoptosis‐inducing ligand.

### 2.2 Investigating tumoritropic migration and tumor killing of canine iNSCs

After confirming transduction and transdifferentiation of canine fibroblasts to iNSCs, in vitro functional assays were conducted to test the migratory and tumor‐killing capability of the new therapeutic cells. The metastatic breast cancer cell line, MDA‐MB‐231‐Br (231‐Br), which was isolated from brain metastases, was selected as the model tumor for in vitro assays because of its ease of growth and sensitivity to TRAIL. Tumor‐homing migration is one of the most critical aspects of stem cell therapy for cancer. To investigate canine iNSC migration, we used real‐time motion analysis and coculture assay systems. Using a two‐well migration insert, the directional migration of iNSCs^TRAIL−/TK−^ toward 231‐Br tumor cells was clearly observed while non‐transdifferentiated fibroblasts moved in a Brownian manner in the 500 μm gap, which similar to our previously published data for mouse and human iNSCs (supp. [Link], [Link]). Next, a coculture assay was used to evaluate the killing capacity of the canine iNSCs lines against 231‐Br cells. The cytotoxicity of iNSC^TRAIL+/TK−^ and iNSC^TRAIL−/TK+^ was tested individually. To explore the efficacy of TRAIL monotherapy, we found that total tumor luminescence decreased as the ratio of iNSCs^TRAIL+/TK−^ to 231‐Br cells increased. A 1:20 ratio of iNSCs^TRAIL+/TK−^ to 231‐Br cells was sufficient to produce a fivefold reduction in tumor viability compared to the 0:1 tumor‐only controls. Promisingly, a 100‐fold reduction in tumor viability was observed when iNSCs were cultured in a 1:1 ratio with 231‐Br cells compared to 0:1 tumor‐only controls following 42 hr of coculture (Figure 1h). Moreover, time‐lapse imaging showed 50% of the 231‐Br cells were dead in less than 10 hr, and nearly 100% of 231‐Br cells are dead in less than 24 hr (supp. Movie S4). Next, exploring the efficacy of TK monotherapy using iNSC^TRAIL−/TK+^ cells, we found a nearly twofold reduction in tumor viability at 72 hr in the 1:20 iNSC:tumor group compared to the 0:1 tumor‐only control group for all samples (Figure 1i). In the absence of TRAIL and VGCV, we showed the iNSCs did not induce apoptosis and allowed the 231‐Br cells to expand (Figure 1j). Overall, these data suggest that canine iNSCs have the ability to seek out tumor cells and induce tumor apoptosis. Importantly, these results also showed similar migratory and killing potencies in all three canine iNSC lines, thus demonstrating the reproducibility of this technology.

### 2.3 Developing methods for MRI tracking of cells

**FIGURE 2:**
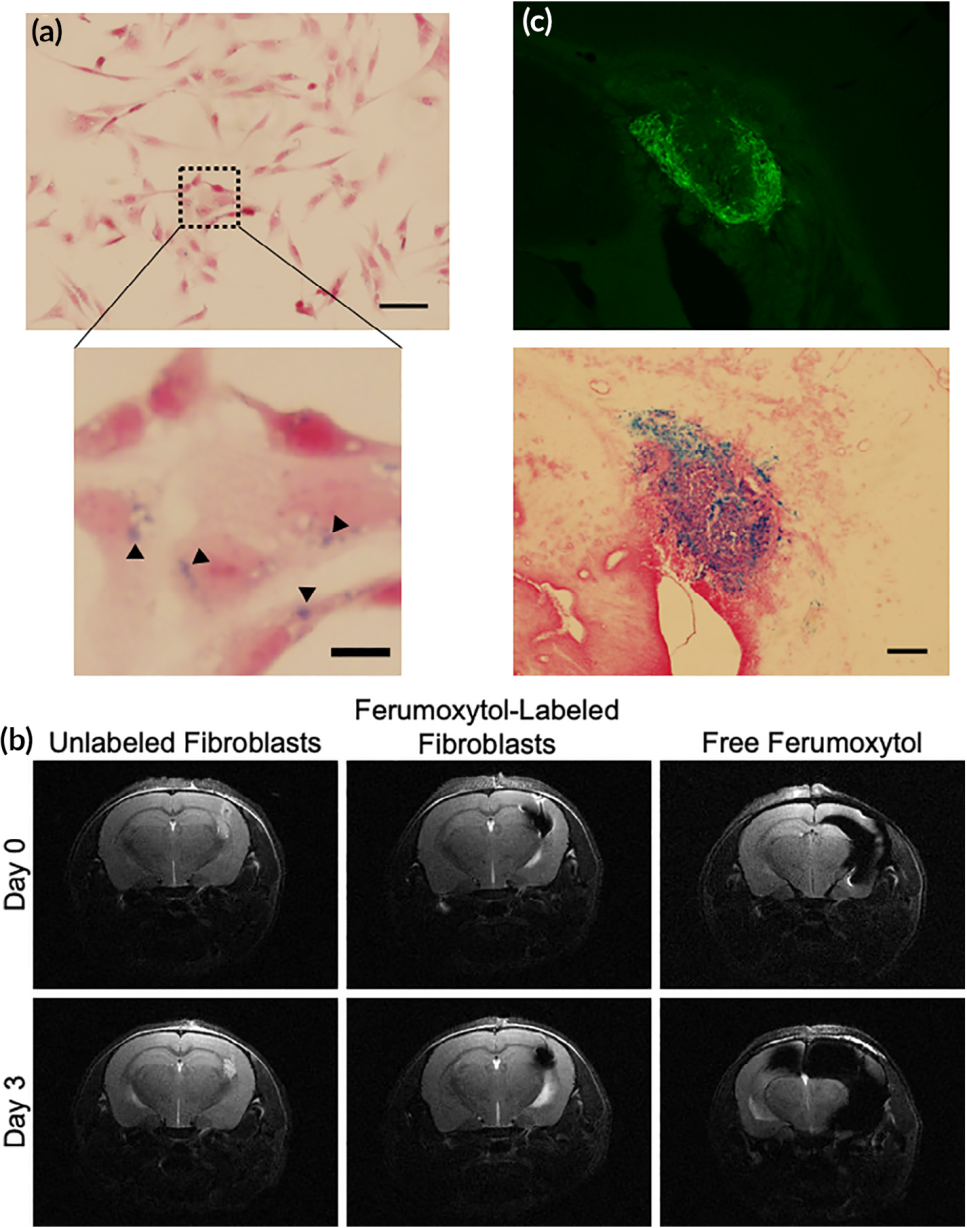
Detection of fibroblasts via magnetic resonance imaging. (a) Prussian blue staining of ferumoxytol‐labeled fibroblasts. Scale bar 50 μm. Magnified image scale bar, 10 μm. (b) Comparison of MR images using unlabeled fibroblasts, ferumoxytol‐labeled fibroblasts, and injection of ferumoxytol suspension over 3 days. (c) Histological sections of mouse brain injected with ferumoxytol‐labeled fibroblasts. Fluorescence (top) depicts fibroblasts, and Prussian blue stain (bottom) depicts ferumoxytol particles. Scale bar 200 μm

Tracking iNSCs in patients is highly beneficial, allowing insights into persistence and distribution. Although fluorescent‐ and bioluminescent‐tracking is valuable in preclinical models, the use of these modalities in human patients is limited by light penetration and other challenges. To generate a method for tracking iNSCs that is compatible with canines and eventual human patients, we opted to label fibroblasts with superparamagnetic iron oxide nanoparticles (ferumoxytol) for MRI. Fibroblasts were used in lieu of iNSCs to expedite this experiment. Ferumoxytol is an FDA‐approved iron replacement therapy and has recently been explored for its use as a cell‐labeling agent.^25^, ^26^, ^27^ Importantly, ferumoxytol has been shown to have no impact on cell viability or stemness, and the iron particles are not readily exocytosed, making it an ideal labeling agent for long‐term tracking of iNSCs.^25^, ^28^ To investigate the potential of labeling fibroblasts for MRI tracking with this agent, the canine cells were transfected with ferumoxytol and assessed for particle uptake in vitro. As shown in Figure 2a, Prussian blue staining of cultured fibroblasts showed homogeneous uptake of iron oxide particles in the fibroblasts. After confirming labeling in vitro, we next assessed imaging and tracking of the labeled cells in vivo. Unlabeled fibroblasts, ferumoxytol‐labeled fibroblasts, and free ferumoxytol were injected into the brain parenchyma of mice. Changes in volumes were then tracked using MRI over 3 days (Figure 2b). Analysis of images showed the cells and free ferumoxytol injection sites are clearly visible in all samples on the day of implant. The unlabeled and iron‐labeled fibroblasts appear confined to a single location in the brain, whereas free ferumoxytol spreads from the injection site. By the third day postinfusion, unlabeled and iron‐labeled fibroblasts remain constrained at their initial injection site, but the free ferumoxytol particles continue to spread medially, caudally, and invade the contralateral hemisphere. To confirm imaging results, mice were sacrificed following MR imaging on day three, brains were harvested, and tissue sections were probed for the presence of iNSCs (Figure 2c). Strong eGFP fluorescence was observed at the site of injection, and Prussian blue staining confirmed the colocalization of iNSCs with iron oxide particles. We believe these studies suggest that we have the ability to isolate and transduce primary canine fibroblasts, transdifferentiate fibroblasts to iNSCs, kill tumor cells using both TRAIL and TK/VGCV, and track iNSCs in mice using MRI. Taken together, these in vitro and murine in vivo studies laid the foundation for the canine scale‐up model. Building on this knowledge, we designed a canine study to evaluate the safety and toxicity of autologous canine iNSCs at low and high doses using two delivery mechanisms anticipated to be used clinically.

### 2.4 Study design to test delivery and dosing

**FIGURE 3:**
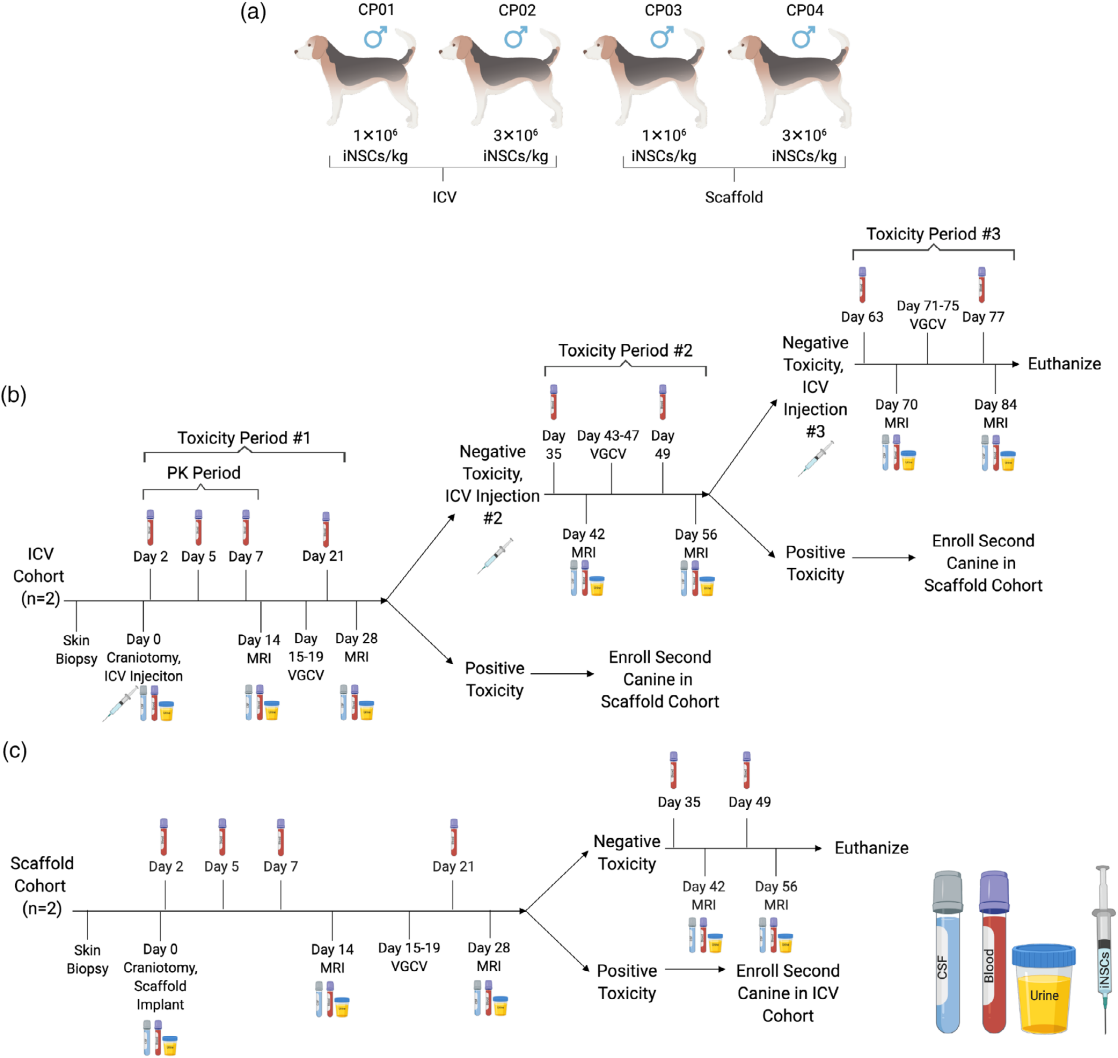
Canine safety and toxicity study design. (a) Intracerebroventricular (ICV) and scaffold treatment arm dosing. (b) ICV study arm timeline. (c) Scaffold treatment arm timeline

In the clinical setting, patient skin biopsies will be collected and converted into iNSCs, which will then be delivered back into patients following surgical resection as part of clinical standard of care for GBM patients. Using our expertise in canine neurosurgery, we created a surgical resection cavity and explored the delivery of iNSCs into the brain using two methods: (a) intracavity seeding within a biocompatible matrix, and (b) infusion into the CSF through a reservoir implanted in the lateral ventricle. Four healthy, male canines were randomized into one of two study arms: ICV injection or scaffold implantation. One canine in each cohort was to receive 1 × 10^6^ autologous iNSCs/kg per dose, and the other canine was to receive 3 × 10^6^ autologous iNSCs/kg per dose. Canines in the ICV cohort, referred to from here on as canine patient 01 and 02 (CP01 and CP02), received a total of three iNSC injections every 4 weeks, while canines in the scaffold cohort, referred to from here on as canine patient 03 and 04 (CP03 and CP04), received a single scaffold implantation (Figure 3a). Animal health and iNSC detection was monitored via routine physical and neurological examinations; blood, urine, and CSF screenings; and MRI. At specified time points, canines were assessed for toxicity. If no toxicity or adverse safety signals were observed, the animal was to continue with the study; however, if a toxicity or safety signal was observed, the second canine in the cohort was redirected to the other study arm (Figure 3b,c).

### 2.5 Manufacturing and scale‐up of canine iNSCs

We next worked on expanding the canine iNSCs manufacturing process. Upon delivery, canines underwent a physical examination to ensure suitability for the study. CP01, CP02, and CP04 weighed 15–20 kg at the time of biopsy. CP03 weighed slightly less at 12 kg. After confirming animal health, full‐thickness skin biopsies were surgically excised and autologous iNSCs were manufactured for each patient. One of the biggest challenges in this study was scaling‐up manufacturing procedures for a canine from a mouse model. As expected, interpatient variability was seen in fibroblast proliferation and transduction efficiency. This made it challenging to predict the iNSC yield after transduction and transdifferentiation. For CP01, we successfully generated the full 1 × 10^6^ iNSCs/kg dose. CP01 received 15 × 10^6^, 14 × 10^6^, and 15 × 10^6^ iNSCs at each dose, respectively. CP02 did not receive the target dose of 3 × 10^6^ iNSCs/kg any of its three doses; CP02 was administered 20 × 10^6^, 17 × 10^6^, and 12 × 10^6^ cells for the first, second, and third doses, respectively. The goal of 1 × 10^6^ iNSC/kg was reached for CP03 and the canine was implanted with a scaffold containing 12 × 10^6^ iNSCs total. CP04 did not reach the dose goal of 3 × 10^6^ iNSCs/kg but was implanted with a scaffold containing 25 × 10^6^ iNSCs total. Further optimization of these processes and incorporation of large‐scale cell culture platforms (e.g., bioreactors) in the future would ameliorate manufacturing scale‐up challenges.

### 2.6 Autologous transplant of canine iNSCs into the brain

**FIGURE 4:**
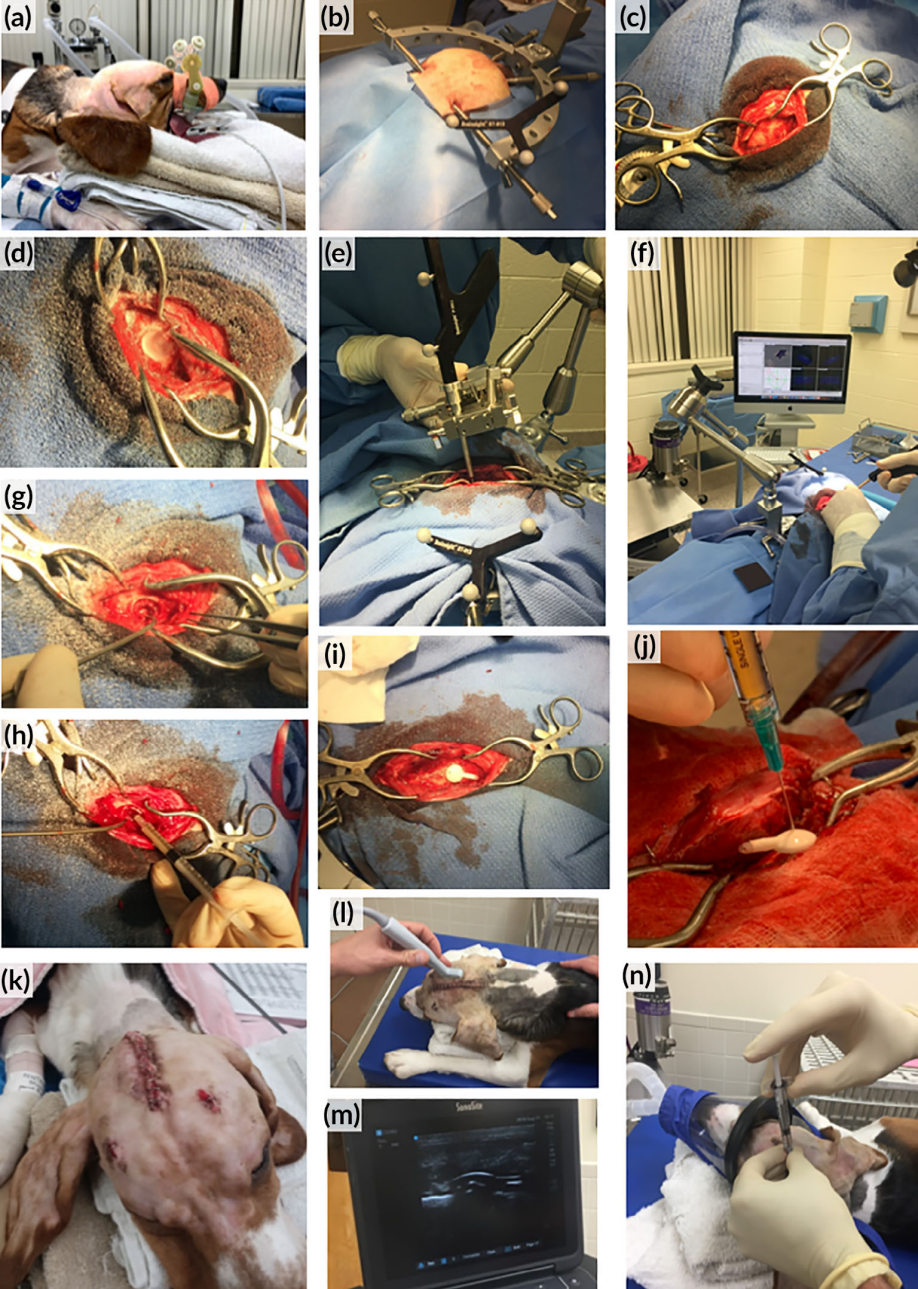
Canine intraoperative procedure. (a) Application of bite plate and attached fiducial markers onto upper dental arcade. (b) Fixation of skull in stereotactic frame and attachment of BrainSight navigation system. (c) Exposing the skull. (d) Craniotomy. (e,f) Using the BrainSight system to locate lateral ventricles. (g) Resection cavity. (h) Injection of iNSCTRAIL+/TK− cells suspended in FLOSEAL. (i) Implantation of intracerebroventricular (ICV) reservoir. (j) Close‐up of injection of iNSCTRAIL+/TK− cells into ICV reservoir. (k) Wound closure. (l) Use of ultrasound to locate ICV reservoir. (m) Sonogram depicting curvature of ICV reservoir. (n) Injection of iNSCTRAIL+/TK− cells into ICV reservoir. iNSCs, induced neural stem cells; TK, thymidine kinase; TRAIL, TNFα‐related apoptosis‐inducing ligand

Brain surgeries are notoriously complex procedures. We sought to closely mirror the surgery and subsequent cell implant in human patients in our canine model. As a first step, we used digital navigation to guide surgery. Canines were anesthetized and custom bite plates with fiducial markers were reaffixed to the canine's maxillary dental arcade (Figure 4a). Due to the conformation of the canine teeth, it was challenging to fit the bite plates in the canines. This risked the accuracy of the surgical navigation. Next, the stereotactic frame was placed around the head of the animal (Figure 4b) and an incision was made to expose the skull (Figure 4c). Using a high‐speed drill, a craniotomy was performed to expose the cerebrum (Figure 4d). The BrainSight system was utilized for neuronavigation using previously obtained MRI scans (Figure 4e,f). Under the guidance of this system, we confirmed key anatomic locations and aspiration was used to remove brain tissue to create a mock resection cavity (Figure 4g). For canines in the scaffold cohort, the iNSC^TRAIL+/TK−^ cells were encapsulated in a FLOSEAL matrix and seeded into the resection cavity (Figure 4h). For the ICV cohort, a separate burr hole was created and an ICV catheter was placed into the ipsilateral (right) lateral ventricle, leaving the reservoir within the skull burr hole below the temporalis muscle and skin (Figure 4i). iNSC^TRAIL+/TK−^ cells were then injected into the reservoir (Figure 4j). Although the lateral ventricle was accessed and CSF obtained in both canines in the ICV cohort, the ICV catheter tip was not precisely placed within the ventricle in either canine due to bite plate inaccuracies and the small size of canine lateral ventricles. Following iNSC^TRAIL+/TK−^ therapy administration, the incisions were closed with sutures and staples (Figure 4k). Canines in the ICV cohort received two subsequent cell doses, given every 4 weeks. For additional doses, the canines were anesthetized, the skin was sterilized, and ultrasound was used to locate the ICV reservoir (Figure 4l,m). The iNSC^TRAIL+/TK−^ cells were administered using a syringe and needle through the skin and muscle without the need to reopen the surgical incision (Figure 4n). Throughout the surgical procedures, special considerations had to be made to maintain sterility while handing off the syringe containing the iNSC^TRAIL+/TK−^ cells. Importantly, no acute injection site reactions were observed in any of the canines following iNSC^TRAIL+/TK−^ administration. VGCV was administered orally to each canine patient 14 days after receiving an iNSC dose for a total of 5 days.

### 2.7 Assessment of cognitive function

All animals underwent neurological examination after recovering from the intracranial surgery. Neurological deficits were limited to visual defects in the eye contralateral to the surgical site in CP01, CP03, and CP04 and transient postural reaction deficits in the contralateral limbs of all canines. These postoperative changes were consistent with the cerebral resection and were not considered a safety signal of the iNSCs.

### 2.8 Tracking persistence of autologous iNSCs

**FIGURE 5:**
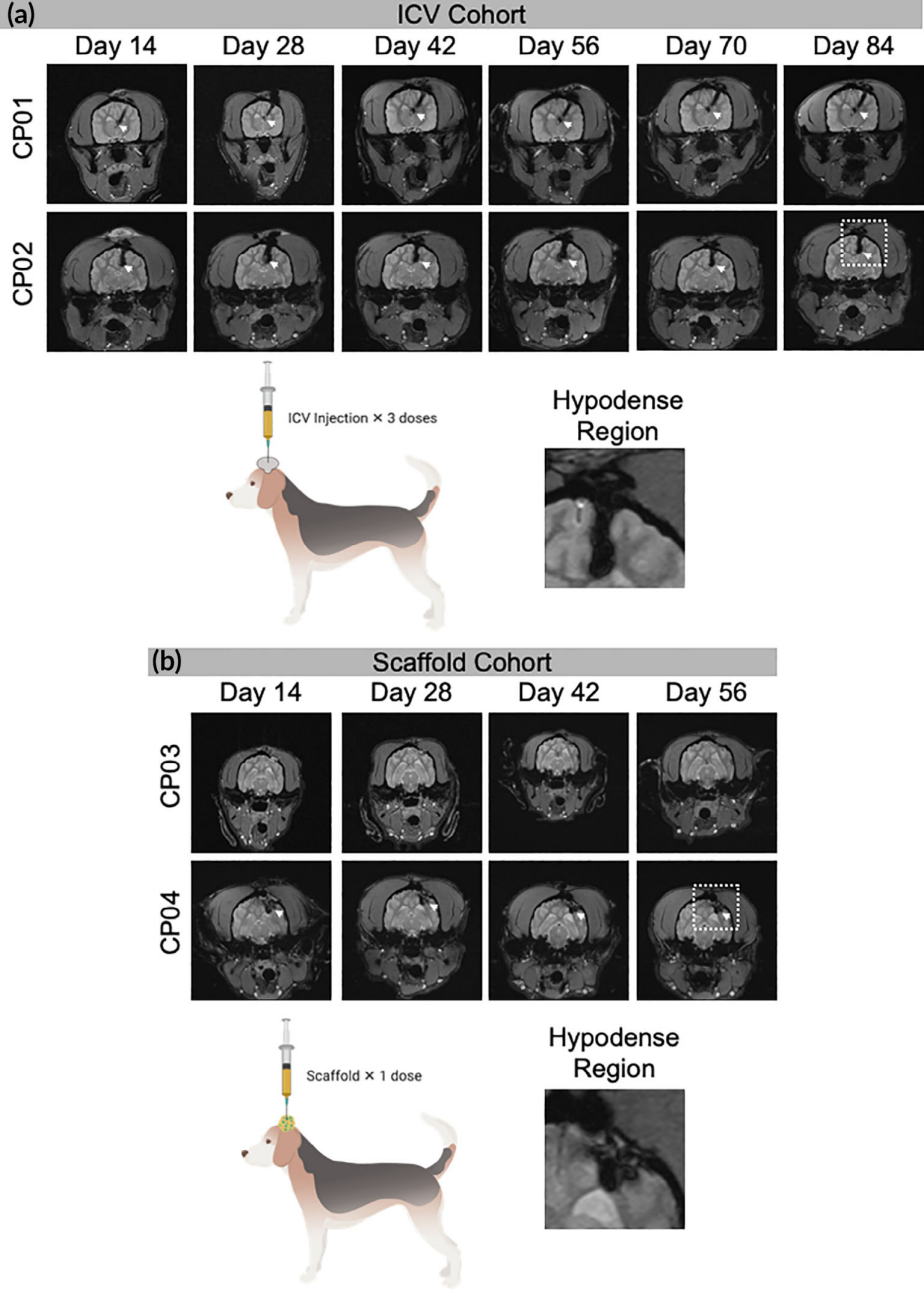
Canine magnetic resonance imaging (MRI). Transverse T2* MRI of canine patients in the (a) intracerebroventricular (ICV) cohort and (b) scaffold cohort. Hypodense regions (white arrows) indicate the presence of blood and/or ferumoxytol. Close up of hypodense region outline by white box

We anticipated the autologous nature of iNSCs would allow the cells to avoid immune rejection, where the longer persistence could offer advantages in efficacy. However, the persistence of personalized iNSC carriers in autologous, immune competent models is unknown. Similar to the proof‐of‐concept murine studies, MRI was used to track iNSC persistence and assess ICV reservoir placement. We first focused on analysis of canines in the ICV group. Interestingly, MRI images from this group suggested that the ICV catheter tip extended beyond the lateral ventricle in the case of CP01 and is positioned slightly dorsomedially to the lateral ventricle in the case of CP02. Despite the partially missed placement, analysis of the MRI images (Figure 5a) showed a hypointense region that remained present through 84 days with no marked reduction in the hypointense signal over time, suggesting iNSCs may persist for several months postinfusion. We next turned our focus to iNSCs delivered via intracavity scaffolds. As shown in Figure 5b, the hypointense regions were less evident for CP03 compared to CP04, likely due to the depth of the resection cavity and/or iNSC dose. However, a hypointense region was clearly visible for CP04 within 14 days postimplant that remained stable and detectable through 56 days posttransplant. While T2* MRI was used to track the presence of iNSCs, it also denotes the presence of blood as it detects the presence of iron in hemoglobin. However, it is expected that blood and its degradation products would resorb over time and a reduction in the hypointense signal would be observed.^29^ As the hypointense region persisted nearly 60 days posttransplant, and without dramatic changes in volume, we believe this suggesting that the hypointense signal is indicative of iNSCs rather than hemoglobin.

### 2.9 Assessing the safety of personalized iNSC therapy

**FIGURE 6:**
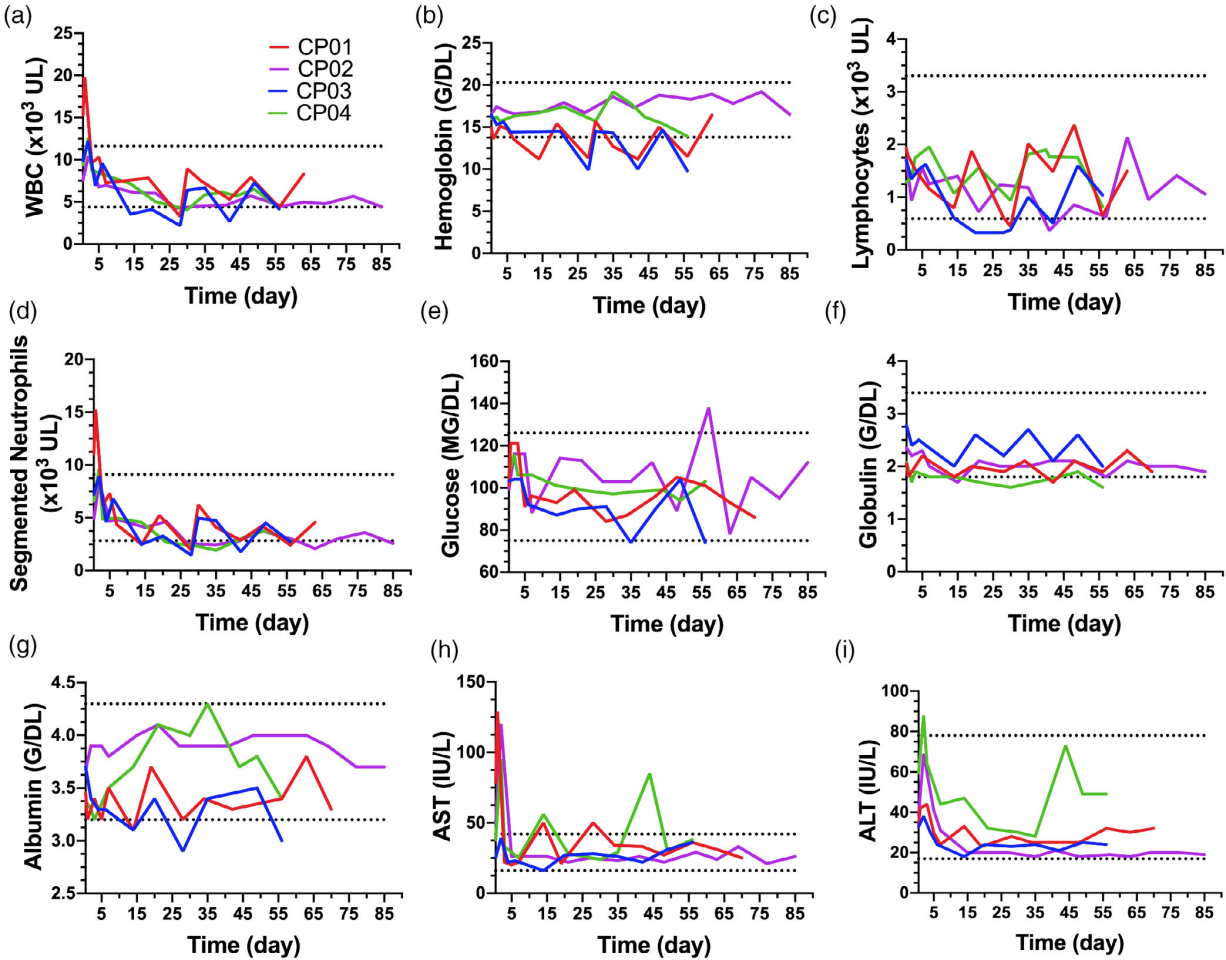
Canine fluid analysis. Select complete blood count and blood chemistry findings, including: (a) white blood cell, (b) hemoglobin, (c) lymphocyte, (d) segmented neutrophil, (e) glucose, (f) globulin, (g) albumin, (h) aspartate aminotransferase (AST), and (i) alanine aminotransferase (ALT) findings. Dotted lines denote normal reference ranges for canines

**FIGURE 7:**
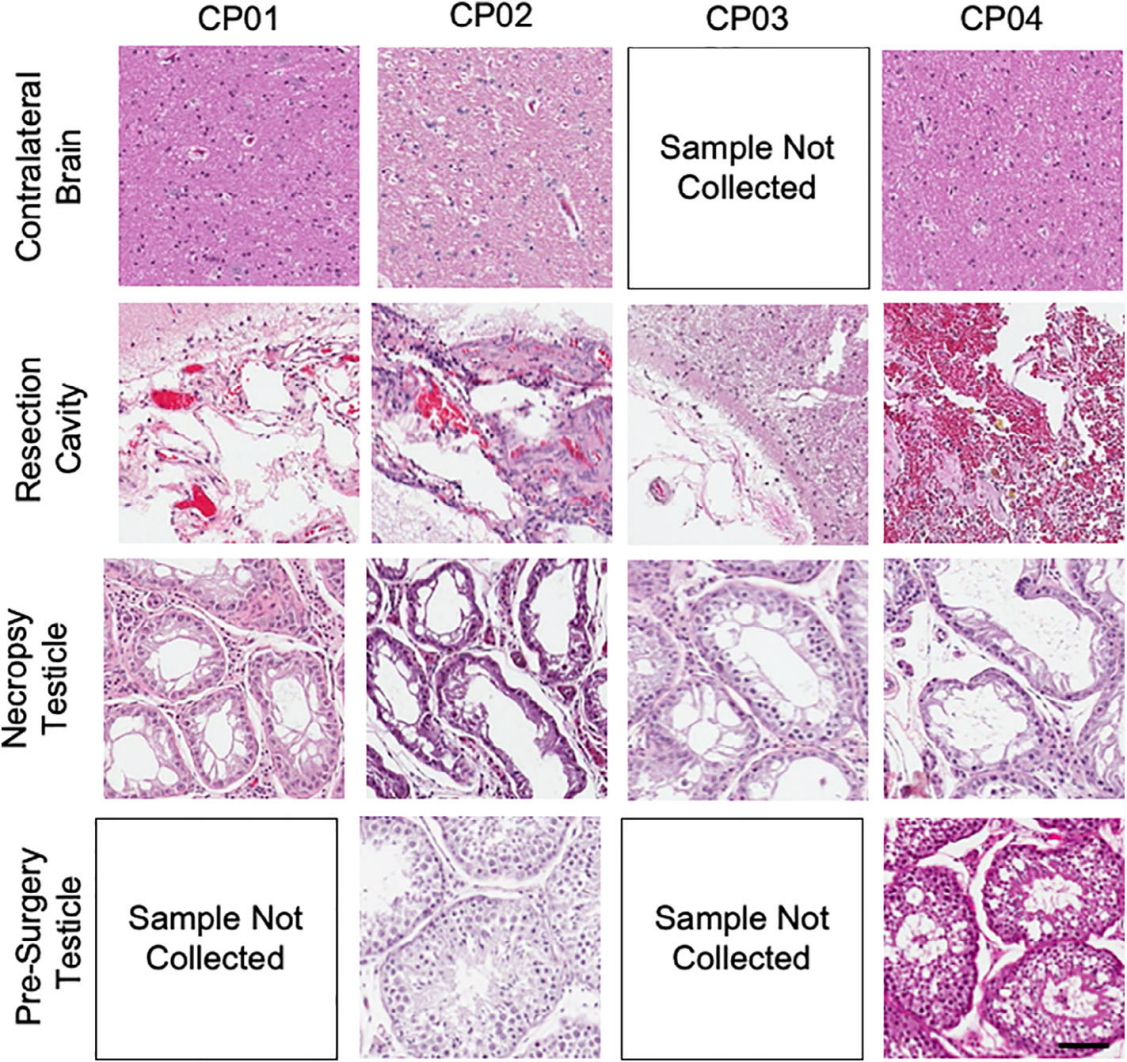
Canine histology. Hematoxylin and eosin staining contralateral brain, resection cavity, testicular tissue at necropsy, and testicular tissue collected presurgery. Scale bar, 100 μm

Demonstrating iNSCs are safe is one of the most important parameters to test as iNSCs move toward human patients. Although human iNSCs have previously been shown to be safe, these studies were limited as they were performed in immune‐depleted mice. The autologous transplant into fully immune‐competent canines offers the potential to more accurately assess iNSC safety in a model mirroring personalized human iNSC therapy. To investigate the safety of iNSCs, canine health was assessed using complete blood count, blood chemistry, urine, and CSF (Figure 6). As anticipated, elevations in white blood cells, segmented neutrophils, aspartate aminotransferase, and alanine aminotransferase were noted immediately following surgery. These elevated levels were attributed to the postsurgical immune response and muscle manipulation during surgery. Interestingly, all canines experienced cyclic neutropenia throughout the study. The observation was first noted in CP01 and CP03 and correlated to MRI days. To ensure the effects were not caused by medications and anesthesia (including IV fluid therapy), peripheral blood was drawn and analyzed in three ways for CP03 on Day 70: (a) prior to any medications or handling, (b) during IV catheter placement after premedication with hydromorphone and midazolam but before anesthesia with propofol and isoflurane, and (c) after receiving all anesthetics and medications and after MRI. All blood draws indicated neutropenia in CP03. For CP02 and CP04, blood was drawn after administration of hydromorphone and midazolam but prior to propofol, isoflurane, and MRI. There was some evidence of dilutional effects of the fluid therapy on blood cell counts. However, surprisingly neutropenia was observed on both MRI and non‐MRI days, unlike in CP01 and CP03. There was also some evidence to suggest that VGCV can cause neutropenia. However, we cannot rule‐out iNSC‐induced neutropenia.^30^ Importantly, urine and CSF findings were unremarkable (data not shown). We also assessed all major organs for any signs of abnormal pathology following necropsy (Figure 7). As anticipated, there were some notable findings near the ICV reservoir implantation site and the scaffold implantation site. These findings included mild to moderate chronic inflammation, mild fibrosis, gliosis, and axon degeneration. Fibrous thickening and chronic inflammation were observed in the meninges in the region of the resection cavities of CP01 and CP02. No abnormalities were identified in the contralateral brain in all canines. However, histological changes in the brain and meninges near the resection cavities in the current study were attributed to postsurgical wound healing. Additionally, histology showed acute spinal cord hemorrhage in all canines, which was attributed to perimortem CSF collection. Observed bone marrow hyperplasia in CP01 was interpreted as a response to the neutropenia detailed above. Spleen, lungs, adrenal glands, liver, and lymph nodes were unremarkable in all canines. However, pathology did note moderate testicular degeneration and aspermia in CP01 and CP03 that was not anticipated. To further investigate this finding, unilateral castrations of canines CP02 and CP04 were performed to determine if testicular findings were age‐, iNSC‐, or drug‐related. No other unanticipated abnormal pathological findings were observed (Figure S1). To further investigate the testicular abnormalities, unilateral castration was performed on CP04's right testis on the day of craniotomy and the left testis was harvested at necropsy. For CP02, unilateral castration of the right testis was performed 15 days after craniotomy; the left testis was harvested at necropsy. The testis of CP02 harvested at necropsy showed marked tissue degeneration. Aspermia in addition to no active spermatogenesis were observed in the testicle harvested at necropsy in CP04. Due to the extent of degeneration observed in the testes of CP01 and CP03 at necropsy, age‐related causes were ruled out. Given the normal histopathology of the presurgery testis in CP02, we attributed the abnormal pathological findings in the testes to the VGCV. GCV and acyclovir have both shown extensive reproductive toxicity in animal models, and the FDA has issued a boxed warning for the reproductive toxicity of VGCV.^31^, ^32^, ^33^ These findings strongly suggest that the abnormal testicular pathology seen in the canines is the result of VGCV and not a direct toxicity of the iNSCs. While this cannot be ascertained for certain without additional control canines, we opted to limit our study to four animals for humane reasons.

## 3 DISCUSSION

NSCs are an emerging new platform for cancer treatment. Their ability to home to distant tumor foci as well as their ability to carry cytotoxic payloads makes them an attractive therapy for GBM. Preclinical studies have demonstrated the efficacy of allogeneic human NSCs and human iNSCs in murine models.^3^, ^4^, ^8^, ^17^ Promisingly, allogeneic NSCs have entered human clinical trials.^34^ However, until now, little data has been published on autologous iNSCs using an animal model that more closely mimics the human condition. To our knowledge, this is the first study to explore the safety, toxicity, and persistence of autologous iNSCs in a large animal model and serves as foundational work for future human clinical trials.

We selected a canine model for several key reasons. First, we required an immune‐competent animal. Previous studies using NSCs have been mostly limited to immunocompromised mice, and therefore lack the ability to identify any adverse reactions or potential safety signals of iNSCs.^3^, ^4^, ^8^ Second, it is well established that murine models are not fully indicative of human conditions. The canine anatomy more closely mimics the spatial scale of humans. Furthermore, unlike murine models, which are often inbred, our canine model allowed us to explore interpatient variability. As anticipated, we observed varying proliferation rates and transduction efficiencies in our canine patient fibroblast lines, and these results mimic what is observed in human patients. Third, we desired an animal model that developed GBM tumors similar to that of humans. While xenograft models of GBM in mice, zebrafish, and drosophila have been studied, canines spontaneously develop glioma lesions with characteristics similar to those of human tumors.^35^, ^36^, ^37^ Again, while this study used nontumor‐bearing canines, this was an important consideration for future research. Combining all of this information, we felt a canine model would be best suited to evaluate the safety of iNSCs and served as the ideal model for forthcoming efficacy studies.

By employing the canine model, we have demonstrated the ability to reproducibly manufacture therapeutic, autologous iNSCs. Using small, full‐thickness skin biopsies, we produced large pools of fibroblasts that could easily be cryostored for future use or immediately transduced and transdifferentiated to become therapeutic iNSCs. Clinically, we envision the process of obtaining skin biopsies to be incorporated into patient standard of care. Overall, while we successfully established personalized canine iNSCs lines, there is room for improvement in cell manufacturing scale‐up. One of the most challenging aspects of this study was culturing millions of cells for each dose. During the cell collection process, we observed entrapment of cells in extracellular matrix nets, and despite trituration and enzymatic digestion, total iNSC yield increased only marginally. Future studies will explore the use of bioreactors to increase culture efficiency and iNSC yield.

Moreover, we also explored two delivery mechanisms to implant iNSCs in the brains of canines. We first investigated intrathecal delivery using a Rickham reservoir. ICV reservoirs are used in patients with GBM to deliver chemotherapeutics into the lateral ventricle. Here, chemotherapy follows the flow of CSF to cover the brain and spinal cord and reaches tumor foci via diffusion. While this system is FDA approved and bypasses the BBB, passive diffusion will prevent the chemotherapy from reaching deep tumor foci.^38^, ^39^ Therefore, we believed that combining the ICV reservoir with our migratory iNSCs would overcome several challenges of current treatment strategies and provide a pathway for future clinical translation. In parallel to ICV reservoirs, we also explored a novel delivery strategy using the FDA approved hemostatic matrix FLOSEAL. FLOSEAL's natural components and hydrogel structure made it a promising, biocompatible choice for delivering iNSCs. We hypothesized that encapsulating iNSCs in FLOSEAL would increase cell persistence, and in future studies, would correlate to increased survival. Our MRI data suggest that FLOSEAL aids in iNSC persistence. Histology further shows no unanticipated brain lesions, demonstrating FLOSEAL's safety. Overall, we found utility in both delivery mechanisms and believe either device could be used to dispense large iNSC doses into human GBM patients.

With the canine model, we were also able to establish the safety of iNSCs by conducting neurological assessments, screening fluids, and analyzing histology. Postural, gait, and vision abnormalities observed in canines mimicked possible adverse reactions during human brain surgery. Additionally, iNSCs did not elicit any concerning safety signals, regardless of ICV reservoir or FLOSEAL scaffold delivery, as determined by blood, urine, or CSF. The transient neutropenia and reproductive toxicity findings were unanticipated, but these could due to administration of the prodrug VGCV rather than the iNSCs.

## 4 CONCLUSION

In conclusion, this research demonstrates the feasibility of manufacturing autologous iNSCs as well as their limited toxicity profile. We further show two clinically translatable methods for delivery. Together, these data support the need for additional large animal efficacy studies and paves the way for future human clinical trials.

## 5 MATERIALS AND METHODS

### 5.1 Tissue harvest and digestion

Skin samples were harvested from canines at North Carolina State University; animals were euthanized for reasons unrelated to this study. Immediately following euthanasia, hair was removed using an electric trimmer. The harvest site was sterilized with betadine followed by 70% ethanol, repeated three times. Full thickness skin samples were acquired using a 6 mm diameter biopsy punch. Biopsies were placed in a 15 ml conical tube containing 10 ml 1X PBS and 2X antibiotic‐antimycotic and transported on ice to the University of North Carolina at Chapel Hill. Skin biopsies were individually transferred into new 15 ml conical tubes each containing 1 ml digestion media (DMEM containing 20% FBS, 1% penicillin–streptomycin, 0.25% collagenase Type I, and 0.05% DNase) and incubated at 37°C/5% CO_2_ overnight. The next day, the conical tubes were vortexed for 20 s to disrupt the skin and disintegrate the dermis. Approximately 7 ml fresh DMEM containing 20% FBS and 1% penicillin–streptomycin was added to the 15 ml tube; and the contents were mixed and transferred to a 10 cm Petri dish. The dishes were returned to the 37°C/5% CO_2_ incubator and left undisturbed for 72 hr.^24^

### 5.2 Fibroblast expansion and cell culture

After 72 hr, media was exchanged for fresh DMEM (henceforth containing 10% FBS and 1% penicillin–streptomycin). Fibroblasts were cultured in this state until the plate reached >70% confluency. Cells were then expanded and subcultured by lifting in 0.05% trypsin–EDTA for 5 min, adding DMEM to neutralize trypsin, centrifuging at 1,000 rpm for 5 min, aspirating supernatant, resuspending in fresh DMEM, and re‐plating in new 15‐cm Petri dishes. Cell strainers (100 μm) were used to separate single cells from residual tissue pieces.

### 5.3 Lentiviral transduction and transdifferentiation

Fibroblasts were cotransduced with lentiviral *rtTA*, *SOX2*, *TK‐mCh*, and *TRAIL‐eGFP* (Iowa University and Duke University Viral Vector Cores). To transduce fibroblasts, cells were incubated with virus and 1 g/ml polybrene overnight at 37°C/5% CO_2_. The following day, the mediate containing virus was aspirated and replaced with fresh DMEM. Cells were monitored for fluorescent signal. Next, to transdifferentiate cells to iNSCs, transduced fibroblasts were plated at a density of 2 × 10^6^ cells per 15 cm Petri dish in DMEM and incubated overnight at 37°C/5% CO_2_. The following day, DMEM was replaced with STEMdiff Neural Induction Medium (STEMCELL Technologies 05835) containing 2 μg/ml doxycycline (NIM + doxy). NIM + doxy media was replaced every other day for a total of 5 days.

### 5.4 Immunohistochemistry

To confirm lentiviral transduction of mCh‐TK and eGFP‐TRAIL vectors into fibroblasts, fluorescence microscopy was conducted prior to transdifferentiation of the cell lines. Cells were monitored daily until maximum fluorescence intensity was observed. Percent fluorescence expression was quantified in ImageJ. To determine extent of transdifferentiation, cells were fixed in 10% formalin for 15 min and washed three times in 1X PBS. Next, cells were incubated in blocking buffer (0.5% bovine serum albumin and 0.25% Triton X‐100 in 1X PBS) for 30 min at room temperature. Primary rabbit anti‐SOX2 (Abcam ab97959) antibody was diluted 1:200 in blocking buffer and incubated for 1 hr at room temperature on a shaker. After three 1X PBS rinses, goat anti‐rabbit Alexa Fluor 488 secondary antibodies (Invitrogen A27034) were diluted 1:600 in blocking buffer and incubated for 1 hr at room temperature on a shaker, protected from light. After three PBS rinses, Hoechst was added to the samples and incubated for 5 min on a shaker, protected from light. Samples were then washed in 1X PBS and imaged. Transduction was deemed successful if >70% of the cells expressed mCh and eGFP, and cells were considered transdifferentiated if >70% of the cells were positive for SOX2.

### 5.5 Iron oxide labeling

On the fifth day of transdifferentiation, iNSCs were labeled with ferumoxytol (AMAG Pharmaceuticals) for MRI imaging. Briefly, 0.0125 mg/ml Lipofectamine 2000 (Invitrogen 11668027) and 0.15 mg/ml ferumoxytol were added to OptiMEM (Gibco), and incubated at room temperature for 1 hr. Next, NIM + doxy media was aspirated from each dish and replaced with 8 ml of the ferumoxytol media. iNSCs were incubated for 4 hr with the ferumoxytol media at 37°C/5% CO_2_. At the completion of 4 hr, media was aspirated, cells were rinsed with 1X PBS, and NIM not containing doxy was added back into the dish. Cells were incubated overnight at 37°C/5% CO_2_.

### 5.6 Cell collection

Following transduction and transdifferentiation, iNSCs were harvested for dosing. First, NIM was aspirated from the dishes. Next, cells were incubated with 4 ml of Accutase (STEMCELL Technologies 07922) at room temperature for 5 min. Dishes were then washed with 4 ml of 1X PBS and passed through 100 μm Falcon Cell Strainers (Fisher Scientific 08‐771‐19). Cells were then pelleted at 1,000 rpm for 5 min. The supernatant was then aspirated, and the cells were resuspended in 1X PBS for counting. To count the cells, a 1:1 dilution was made in Trypan Blue (Invitrogen T10282), and the cells were quantified by a Countess II Automated Cell Counter (Invitrogen). For canine studies, cells were transported on ice to North Carolina State University, and at the time of craniotomy, cells that were to be injected ICV were resuspended in 600 μl of artificial CSF (TOCRIS 3525).

### 5.7 In vitro cell migration

Two‐well inserts (Ibidi 80209) were placed in dry tissue‐culture treated six‐well plates. Then, 8 × 10^4^ MDA‐MB‐231Br‐mCh‐Fluc cells (gift from Dr Toshiyuki Yoneda, University of Texas Health Science Center at San Antonio) were seeded in DMEM in one well of the insert and 4 × 10^4^ canine iNSCs or control canine fibroblasts were seeded in DMEM in the second well of the insert. Cells were incubated overnight at 37°C/5% CO_2_. The following day, DMEM was aspirated and the two‐well insert was removed. The cells were then incubated in NIM and serial fluorescence imaging (EVOS FL Auto) was performed over 66 hr to assess migration.

### 5.8 In vitro tumor killing

MDA‐MB‐231‐Br‐mCh‐Fluc cells and canine iNSC^TRAIL+/TK−^ were cocultured in NIM media at ratios of 0:1, 1:20, 1:10, 1:4, and 1:1, respectively. Serial fluorescence imaging (Molecular Devices SpectraMax M2) was performed over 24 hr to assess tumor viability. To activate TK, 20 μg/ml GCV was added to the culture. Images were analyzed using ImageJ by taking a measurement of the average gray value, where an increase in the average gray value would indicate cell proliferation. These results were verified via bioluminescence imaging (BLI). Using 0.15 mg d‐luciferin (PerkinElmer 122799) in 1X PBS, BLI was quantified at 42 and 72 hr for iNSC^TRAIL+/TK−^ and iNSC^TRAIL+/TK+^ killing assays, respectively.

### 5.9 Scaffold preparation and cell seeding

iNSCs were prepared as described above. FLOSEAL kits (Baxter 1503350) and components were prepared according to manufacturer's instructions. Briefly, lyophilized thrombin was reconstituted in 200 mM CaCl_2_. The prepared thrombin solution was used to resuspend the iNSC cell pellet, and the cell/thrombin solution was loaded into the provided female Luer connector syringe. Next, the gelatin matrix was loaded into the Luer Lock syringe; the syringe was sterilized via UV light. Following sterilization, the syringes were connected head‐to‐head and passaged 20 times to thoroughly mix. The iNSC‐containing FLOSEAL matrix was kept on ice until use.

### 5.10 Murine in vivo surgical procedure

All murine procedures were approved by the Animal Care and Use Committee at the University of North Carolina at Chapel Hill. Then, 6–8‐week‐old female nude athymic nude mice (Animal Studies Core, University of North Carolina‐Chapel Hill) were anesthetized using 2.5% inhaled isoflurane and stabilized in the prone position in a stereotactic frame. The surgical site was sterilized using 70% isopropyl alcohol and betadine. Next, a midline incision was made on the mouse's head to expose the skull, and the underlying fascia was removed using a cotton swab. Then a burr hole was created with a microdrill. Using a Hamilton syringe, 1 × 10^6^ unlabeled canine fibroblasts or ferumoxytol‐labeled fibroblasts were injected into the brain parenchyma at 1 μl/min. Total volume of 9 μl of ferumoxytol was used as a positive control. Following injection, the syringe was left untouched for 5 min to prevent reflux. The syringe was then removed slowly, and the incision was closed with Vetbond tissue adhesive (3M 1469SB). Postoperative pain was managed with 5 mg/kg meloxicam.

### 5.11 Murine MRI

T2 rapid acquisition with relaxation enhancement MR images were taken using a Bruker 9.4 T BioSpec scanner. A 72 mm quad‐volume coil (Bruker Biospin) was used for radiofrequency transmission. Images were acquired using a repetition time of 10,000 ms, echo time of 8 ms, 15 averages, 51.05 Hz bandwidth, 0.75 mm slices, and 0.1 × 0.1 × 0.75 mm resolution. Mice were anesthetized with 3% inhaled isoflurane.

### 5.12 Murine organ harvest and tissue processing

Mice were anesthetized with 5% isoflurane. Cardiac perfusion was done using 5 ml 1X PBS followed by 5 ml 10% formalin into the left ventricle of the heart. Extracted brains were incubated in 10% formalin overnight. The next day, brains were incubated in 30% sucrose in 1X PBS at 4°C until the tissue sank. Brains were then mounted using OCT compound, cut into 40 μm sections, and analyzed as floating sections. To detect the presence of ferumoxytol‐labeled iNSCs, tissues were stained with Prussian blue following manufacturer's instructions (Eng Scientific Inc. 3160).

### 5.13 Canines

Four 1–2‐year‐old, purpose‐bred male beagles were obtained from a U.S. Department of Agriculture Class A vendor (Oak Hill Genetics). The weights of CP01‐CP04 were 14.5, 17.0, 9.9, and 14.8 kg, respectively, prior to surgery. Canines were co‐housed in groups of 2, fed a commercial diet twice daily, and provided water ad libitum. Physical and neurological examinations, a complete blood count, serum biochemistry, and urinalysis were performed at admission to confirm clinically healthy status. All canine procedures were approved by the Institutional Animal Care and Use Committee at North Carolina State University.

### 5.14 Canine anesthetic and sampling procedures

Canines were anesthetized for MRI, CSF collection, and surgical procedures. Canines were premedicated with hydromorphone (0.1 mg/kg intramuscularly [IM]) and midazolam (0.25 mg/kg IM) and anesthesia was induced with propofol (4 mg/kg intravenously [IV] to effect) and maintained with isoflurane in oxygen (1–3% to effect). CSF samples were collected from the cerebellomedullary cistern. Urine samples were collected by urethral catheterization. Blood, urine, and CSF samples were collected periodically from the canines before and after iNSC implantation.

### 5.15 Canine iNSC production

The skin of the dorsal cervical region was prepared as described above and 1 × 3 cm full thickness, rectangular skin biopsies were obtained and placed into transport media on ice. iNSCs were manufactured as described above. For postoperative analgesia, canines were given carprofen (4.4 mg/kg SQ followed by 4.4 mg/kg PO q 24 hr for 3 days).

### 5.16 Canine MRI

MRI was conducted using three different systems as the study overlapped with the decommissioning of an older MRI unit (Siemens 1.5 Tesla Symphony), installation of an upgraded system (Siemens 3.0 Tesla Skyra), and use of a “bridge” unit (Siemens 1.5 Tesla Symphony equipped with older software). Sequences obtained included T1‐weighted fast spin echo (FSE), T2‐weighted FSE, T1‐ and T2‐weighted fluid attenuated inversion recovery, gradient echo (T2*), and susceptibility‐weighted imaging. T1‐weighted images were also obtained after administration of Gadoversetamide (0.1 mmol/kg, Optimark, Mallinckrodt Inc., St. Louis, MO). For the initial MRI studies, a T1‐weighted, 3D volumetric sequence was also obtained (MP‐RAGE) for use with a veterinary neuronavigation system (BrainSight, Rogue Research, Montreal, Canada). Bite plates affixed with MRI‐compatible fiducial markers were also utilized in this initial MRI study as a prelude to neuronavigation and were created using Hydroplastic impression material (Tak Systems, Wareham, MA).

### 5.17 Canine craniectomy and iNSC implantation

In preparation for craniectomy, canines were anesthetized as described above except that a continuous infusion of propofol (200 μg/kg/min) was utilized to decrease isoflurane requirements. Canines were mechanically ventilated; the head was shaved and surgically prepared and placed in a head frame for neuronavigation (BrainSight, Rogue Research). The bite plate made prior to the first MRI was replaced and used to register the canine for neuronavigation. The iNSCs were delivered in a FLOSEAL scaffold in two canines and through an intraventricular catheter in the other two canines. To simulate a resection cavity remaining after tumor removal, a craniectomy was created using a high‐speed drill followed by durotomy. A 2 × 2 cm region of cerebral cortex and underlying white matter was removed, centered on the marginal gyrus near the junction of the parietal and occipital lobes. In CP03 and CP04, the iNSC‐FLOSEAL scaffold was injected into the resection cavity; in the canines with intraventricular catheters, the resection cavity was left unfilled. The dura was then manually apposed and covered with an artificial dural product (DuraGen Plus, Integra LifeSciences, Plainsboro, NJ). Neither the dura nor the craniectomy defect were primarily closed. In CP01 and CP02, an intraventricular catheter was placed into the right lateral ventricle after creation of a 6‐mm burr hole. The catheter was attached to a Rickham reservoir that was seated into the burr hole to create a ventriculostomy system (Codman Holter Rickham reservoir, Integra LifeSciences). The temporalis muscle, subcutaneous tissues, and skin were closed using suture and the skin was closed using staples. A fentanyl patch (50–75 μg/hr) was placed immediately following surgery, and hydromorphone (0.1 mg/kg IV q 6 hr) was administered until the patch took effect approximately 12 hr after placement. For CP01 and CP02, ultrasound navigation was used to inject the second and third iNSC doses into the Rickham reservoir.

### 5.18 Canine castration

In order to further investigate potential reproductive toxicity, CP02 and CP04 underwent unilateral castration for histopathological analysis and sperm evaluation. These procedures were performed using standard techniques and the contralateral testis was similarly evaluated at the time of autopsy.

### 5.19 Canine postoperative procedures and monitoring

Following intracranial surgery, all canines underwent a neurological exam to assess gait, motor function, postural reactions, segmental spinal reflexes, and cranial nerve function. Blood samples were collected from the ICV cohort at baseline prior to surgery, every other postoperative day through Day 7, and then once per week through week 12. The scaffold cohort had blood drawn at baseline, every other postoperative day through Day 7, and then once per week through Week 8. Urine and CSF were collected from the ICV cohort at baseline and every 2 weeks through Day 84. The scaffold cohort had urine and CSF collected at baseline and every 2 weeks through Day 56. All animals were routinely monitored for adverse events, pain, and signs of infection. The fentanyl patch, which was applied immediately following during surgery, was removed after 5 days. VGCV was administered 2 weeks following scaffold implantation and 2 weeks after each ICV dose at 450 mg PO q 5 days.

### 5.20 Canine euthanasia and necropsy

At the study endpoint, all canines were euthanized via an intentional sodium pentobarbital overdose at 85 mg/kg. Necropsy was performed immediately after euthanasia. Tissue samples were fixed in 10% formalin overnight in preparation for histological analysis.

### 5.21 Canine histology

Hematoxylin and eosin staining was performed on 5 μm sections of formalin‐fixed, paraffin‐embedded tissue samples. Histopathological analysis was performed by board‐certified veterinary pathologists (L. B. B., D. A. T.).

### 5.22 Statistical analysis

Data are expressed as mean ± *SD*.

## CONFLICTS OF INTEREST

K. T. S. and S. D. H have an ownership interest in Falcon Therapeutics, Inc., which has licensed aspects of iNSC technology from the University of North Carolina at Chapel Hill. H. N. B., A. V., S. K., C. L. M., L. R., L. B. B., and D. A. T. have no conflicts to disclose.

## AUTHOR CONTRIBUTIONS

Hunter N. Bomba and Kevin T. Sheets wrote the manuscript. Kevin T. Sheets and Shawn D. Hingtgen designed and analyzed murine experiments, and Kevin T. Sheets conducted murine experiments. Kevin T. Sheets, Simon Khagi, Christopher L. Mariani, Laura Ruterbories, and Shawn D. Hingtgen designed canine study. Christopher L. Mariani and Laura Ruterbories performed canine surgical procedures, monitored animals, and collected tissue and fluid samples. Hunter N. Bomba, Kevin T. Sheets, and Alain Valdivia prepared canine iNSCs. Luke B. Borst, Christopher L. Mariani, and Debra A. Tokarz performed necropsies and analyzed canine histology samples. Simon Khagi procured ICV reservoirs and scaffold material. All authors reviewed and edited manuscript.

## Supporting information


**Figure S1** Canine Histology. Representative histological section of canine spleen, liver, lung, lumbar spinal cord, and bone marrow. Scale bar 200 μm.

**Movie S1** Brownian Motion of Fibroblasts. Scale bar 200 μm.

**Movie S2** Directional Migration of iNSCs to 231‐Br Tumor Cells. Scale bar 200 μm.

**Movie S3** Non‐direction Migration of Fibroblasts to 231‐Br Tumor Cells. Scale bar 200 μm.

**Movie S4** Coculture of iNSC^TRAIL+/TK−^ cells with 231‐Br Tumor Cells. Scale bar 200 μm.
