# Supplementary figures and images for: Personalized‐induced neural stem cell therapy: Generation, transplant, and safety in a large animal model
Source: Bioeng Transl Med. 2020 Jul 15;6(1):e10171. doi: 10.1002/btm2.10171 (PMC7823134; doi:10.1002/btm2.10171)

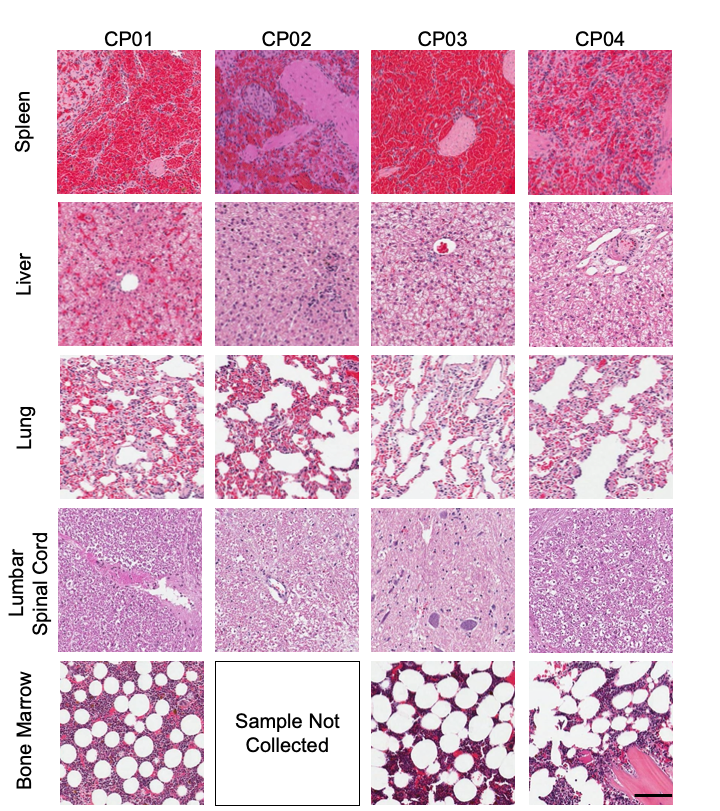

Supplement: Supplementary file 1 — Figure S1 Canine Histology. Representative histological section of canine spleen, liver, lung, lumbar spinal cord, and bone marrow. Scale bar 200 μm. [file BTM2-6-e10171-s001.tiff]
